# Advancement of Polyaniline/Carbon Nanotubes Based Thermoelectric Composites

**DOI:** 10.3390/ma15238644

**Published:** 2022-12-04

**Authors:** Chun Zhang, Hui Li, Yalong Liu, Pengcheng Li, Siqi Liu, Chaobin He

**Affiliations:** 1 Hubei Key Laboratory of Plasma Chemistry and Advanced Materials, Hubei Engineering Technology Research Center of Optoelectronic and New Energy Materials, School of Materials Science and Engineering, Wuhan Institute of Technology, Wuhan 430205, China; 2 Department of Materials Science & Engineering, National University of Singapore, 9 Engineering Drive 1, Singapore 117574, Singapore; 3 Institute of Materials Research and Engineering, A*STAR (Agency for Science, Technology and Research), Singapore 117602, Singapore

**Keywords:** thermoelectric, polyaniline/carbon nanotubes composites, microstructure, electrical conductivity, Seebeck coefficient

## Abstract

Organic thermoelectric (TE) materials have been widely investigated due to their good stability, easy synthesis, and high electrical conductivity. Among them, polyaniline/carbon nanotubes (PANI/CNTs) composites have attracted significant attention for pursuing enhanced TE properties to meet the demands of commercial applications. In this review, we summarize recent advances in versatile PANI/CNTs composites in terms of the dispersion methods of CNTs (such as the addition of surfactants, mechanical grinding, and CNT functional group modification methods), fabrication engineering (physical blending and in-situ polymerization), post-treatments (solvent treatments to regulate the doping level and microstructure of PANI), and multi-components composites (incorporation of other components to enhance energy filtering effect and Seebeck coefficient), respectively. Various approaches are comprehensively discussed to illustrate the microstructure modulation and conduction mechanism within PANI/CNTs composites. Furthermore, we briefly give an outlook on the challenges of the PANI/CNTs composites for achieving high performance and hope to pave a way for future development of high-performance PANI/CNTs composites for sustainable energy utilization.

## 1. Introduction

With the dramatically increasing consumption of fossil fuels, the crisis of resource depletion and environmental pollution becomes more urgent. Fossil fuels generate a lot of heat during their utilization, and most of this heat is directly emitted into the atmosphere causing energy waste. If the waste heat can be collected and reused reasonably, the energy utilization efficiency can be greatly improved, which will effectively alleviate the energy crisis and environmental problems [1,2,3]. Therefore, green energy and energy conversion techniques have attracted increasing attention in recent years. Thermoelectric (TE) materials, which can directly convert heat energy into electricity via the Seebeck effect, are considered one of the most promising candidates for achieving sustainable energy utilization [4]. Currently, great efforts have been devoted to developing versatile high-performance TE materials to promote their commercial applications in electronics, the automotive industry, medical sensing, and other fields [5,6,7,8,9].

Generally, the TE power generation efficiency is mainly determined by the dimensionless figure of merit (*ZT*) given by

(1) $$ZT=\frac{S^{2}\sigma }{\kappa }T$$

where *S* is the Seebeck coefficient, *σ* is the electrical conductivity, *T* is the absolute temperature, and *κ* is the thermal conductivity, respectively [10,11]. Preeminent TE materials exhibit high electrical conductivity (*σ*), high Seebeck coefficient (*S*), and low thermal conductivity (*κ*) [12,13]. Since the thermal conductivity of organic TE materials is relatively low and not easily available, the power factor (PF, defined as *S*^2^*σ*) is usually used to evaluate the performance of organic TE materials [14]. The electrical conductivity can be expressed by the following equation:

(2) σ=nμe

where *n*, *μ*, and *e* represent the carrier concentration, carrier mobility, and electron charge, respectively [14,15,16]. From Equation (2), it can be seen that the carrier concentration and mobility have a great effect on the *σ* of the material, and *σ* is increased with increasing of *n*. *S* is presented as an equation:

(3) $$S=\frac{8\pi^{2}k_{B}^{2}}{3eh^{2}}m^{*}T{\left(\frac{\pi }{3n}\right)}^{2/3}$$

where k_B_, h, m^*^, and *n* are the Boltzmann constant, the Planck constant, the effective mass, and carrier concentration, respectively [17]. According to Equation (3), it is necessary to increase the effective mass of carriers or reduce the concentration of carriers for pursuing high *S*. Unfortunately, two parameters of *σ* and *S* always have a strong inverse relationship, resulting in the difficulty in the maximization of TE performance [18,19]. Therefore, it is crucial to tune carrier concentration properly with a compromise of *S* with *σ* [20], which is strongly dependent on the microstructure of the composites.

**Figure 1:**
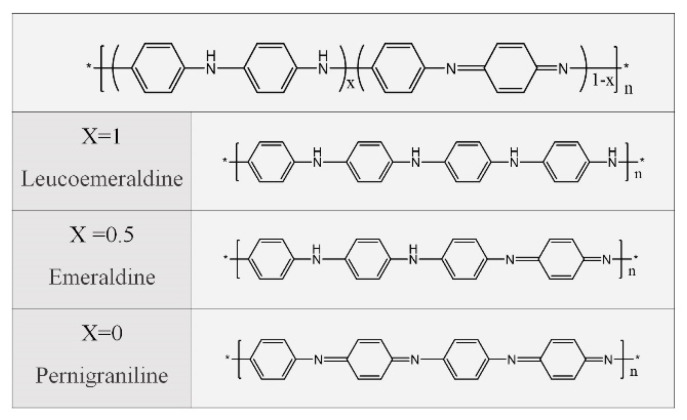
Three common forms of PANI.

Recently, most of the research has focused on inorganic semiconductors, such as Bi_2_Te_3_, PbTe, SnSeS, etc. [21,22,23,24,25,26], and their TE performance has been gradually improved to fulfill the requirements of practical applications. However, the processing difficulties, high cost, brittleness, and toxicity restrict their further applications, especially for flexible and wearable systems [27,28]. Compared with inorganic TE materials, organic materials, including polyacetylene, poly(3,4-ethylenedioxythiophene), polypyrrole, polyaniline (PANI), and so on, have advantages in the wearable electronic field [29,30,31,32,33,34,35]. In particular, PANI has gained special interest among conductive polymers due to its good processability, environmental stability, economic feasibility, low thermal conductivity, and tunable electrical properties [36]. The PANI molecule consists of benzene and quinone rings connected by N atoms. The *σ* of PANI is determined by its structure and doping level [37]. As shown in Figure 1, when X = 1, 0.5, 0, PANI is in the reduced, intermediate oxidation, and oxidation states respectively. When X = 0.5, it is a p-type semiconductor with many conjugated *π*-electrons on the main chain [36]. The *π-π* conjugation between the benzene and quinone rings is the main tunnel for carrier transport. With a proper doping process, carriers are carried out by intra- and inter-chain hopping in the PANI molecules [36,38], contributing to efficient carrier transport and thus enhanced *σ*. Though *σ* up to 300 S cm^−1^ has been achieved when doped with camphor sulfonic acid (CSA), its moderate *σ* is still a great challenge for efficient TE conversion and further commercial applications [39,40]. Therefore, numerous efforts are required to improve the TE performance of PANI-based materials.

To date, versatile PANI-based composites have been developed with the incorporation of nanomaterials that possess high *σ* or *S*. A variety of nanomaterials, such as carbon nanomaterials, inorganic TE materials, and other conductive polymers, have been widely studied and summarized as effective fillers to develop PANI-based composites with enhanced TE properties [41,42,43]. Due to extremely high electrical and mechanical behaviors, carbon nanotubes (CNTs) have attracted significant interest in polymer-based composites [44,45,46]. The PANI/CNTs composites exhibit greatly enhanced *σ* and *S* synergistically compared with the pure components. This is ascribed to the strong *π-π* conjugation interactions between CNTs and PANI to form ordered PANI chains on the surface of CNTs, as well as the energy filtering effect at the interfaces between CNTs and PANI to selectively filter out low-energy carriers [47]. Currently, numerous strategies have been developed to fabricate PANI/CNTs composites and modulate their morphology, enabling PANI/CNTs composites to be promising materials for sustainable energy harvesting.

In this review, we briefly summarized the recent progress of PANI/CNTs composites and focused on the fabrication process to modulate morphology and thus optimize their TE performance. Versatile PANI/CNTs composites were discussed in terms of dispersion strategies of CNTs, fabrication engineering, post-treatments, and multi-component hybrid composites. First, as the dispersibility of CNTs has a strong influence on charge carrier transport, varied approaches toward uniform dispersion of CNTs have been widely investigated to reduce CNTs aggregates. Dispersion approaches of CNTs were summarized, enabling CNTs to be uniformly distributed within PANI/CNTs composites. Then, various PANI/CNTs composites were prepared mainly through physical blending and in-situ polymerization methods, and the resultant composites could be employed as-prepared or after secondary doping. Particularly, the microstructure of the composites could also be tuned by modulating the polymerization process of PANI. Furthermore, post-treatment is also an effective strategy to optimize carrier transport and improve their TE properties. In order to enhance the energy filtering effect, other components were usually incorporated into the composites to further enhance the TE performance. In this review, the recent development of PANI/CNTs composites about preparation strategies, microstructure modulation, fundamental mechanisms, and TE properties are comprehensively described to illustrate the optimization of their morphology and TE properties. Through the introduction of various approaches to modulate fabrication processing and microstructure, the development of high-performance PANI/CNTs composite materials was systematically clarified, paving a way for their practical application in sustainable energy harvesting.

## 2. Dispersion Strategies of CNTs

Due to the strong Van der Waals interactions between CNTs, CNTs tend to form agglomerations which result in deteriorated carrier transport and electrical properties of the composites [48,49]. Tremendous efforts have been devoted to dispersing CNTs in the solvent and polymer matrix uniformly. Various methods, such as the addition of surfactants, mechanical stirring and grinding, ultrasonic dual mixing technique, and functional group modification, have been widely applied to CNTs to improve their dispersibility [50,51,52,53,54,55,56], thereby enabling the resultant nanocomposites to possess prominent mechanical, viscoelastic, and conductive properties.

**Figure 2:**
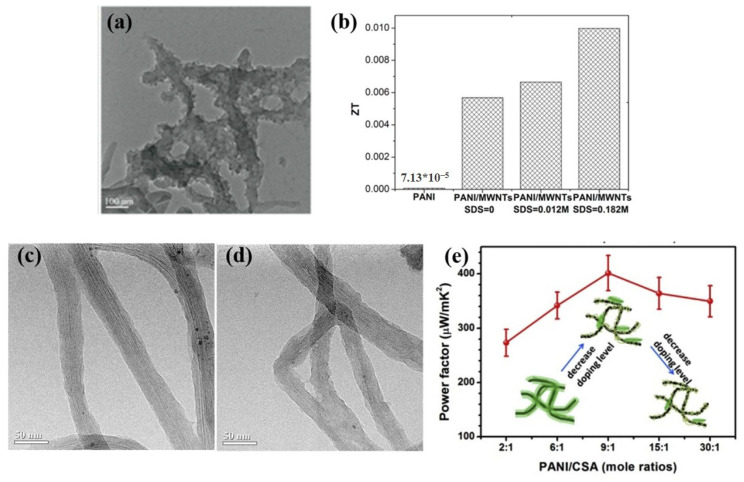
(**a**) TEM images of porous PANI-coated MWNT (ρSDS = 0.182 M) [57]; (**b**) Histograms of *ZT* of PANI and PANI-coated MWNTs with ρSDS = 0, 0.012 M, and 0.182 M, respectively [57]; TEM images of 57 wt% (**c**) A-CNT/PANI and (**d**) U-CNT/PANI [59]; (**e**) Corresponding PF of 94 wt% A-CNT/PANI composites with varying mole ratios of PANI/CSA [59].

Zhang et al. [57] prepared sodium dodecyl sulfate (SDS)-wrapped porous MWNTs (core)/PANI (shell) nanostructured composites by in situ polymerization. The TEM image showed that the MWNTs were more uniformly dispersed in PANI with the addition of SDS, and the surface of PANI was porous and rough (Figure 2a). The *S* of the composite was improved by thermal annealing. The optimal *S* of the PANI-coated MWNT (ρSDS = 0.182 M) composite reached 79.8 µV K^−1^ and the *ZT* was 0.01 at room temperature (Figure 2b). Compared with PANI-coated MWNT without surfactant (ρSDS = 0), the *ZT* of PANI-coated MWNT (ρSDS = 0.182 M) was about twice that of PANI-coated MWNT (ρSDS = 0). Moreover, Zhang et al. [58] prepared nanotube composites with well-dispersed MWCNTs in the PANI matrix using a low-temperature grinding method. The uniform dispersion of CNT promoted the formation of a good interconnected conductive network in the composites, which contributed to enhanced carrier transport and electrical properties of composite materials.

In addition, surface modification of CNTs is another effective strategy to improve its dispersibility [60,61]. Liu and Yu [62] reported non-covalent functionalized MWNTs with polypyrrole (PPy-MWNTs) to synthesize PANI/PPy-MWNTs composites by in situ polymerization. The surface-functionalized PPy nanolayer on the MWNTs effectively avoids the formation of CNTs agglomerates, resulting in good MWNTs dispersion in PANI/PPy-MWNTs composites. While MWNTs clusters were observed in the r-MWNT/PANI composites without PPy functionalization. Meanwhile, PPy-MWNTs were conducive to the enhanced PANI-ordered structure, which could facilitate charge transport. At 28.6 wt% MWNT loading, the *σ* and *S* of the prepared composites were significantly increased, reaching a maximum PF of 3.1 µW m^−1^K^−2^ at room temperature. Li et al. [59] prepared amine-functionalized SW/DWCNT (A-CNT)/PANI composites with high *σ* and *S* by in situ polymerization. The amino functional group on the CNT allowed good wettability and dispersibility in the solution, enabling the PANI to be uniformly coated on the A-CNT surface. This facilitates the formation of highly conductive networks in the composites. While PANI nodules were observed in the unmodified carbon nanotubes (U-CNT)/PANI, which inhomogeneously coated on the surface of U-CNT as shown in Figure 2c,d. Due to the unique *π-π* interaction between PANI and A-CNT, as well as the homogeneous conductive microstructure, the effective charge transfer within the composite was promoted. The composite achieved a PF of 401 µW m^−1^K^−2^ at a doping ratio of PANI/CSA = 9:1 at room temperature (Figure 2e).

Despite the dispersion of CNTs can be enhanced by utilizing surface activators and functionalization, the surface activators are not conductive and the functionalization process may disrupt the orderliness of CNTs and affect their charge conduction. Therefore, further investigations about the dispersion of CNTs are required to simultaneously enhance the dispersibility and conductivity of CNTs, promoting the TE properties of CNT-based composites.

## 3. Preparation Engineering

### 3.1. Physical Blending

**Figure 3:**
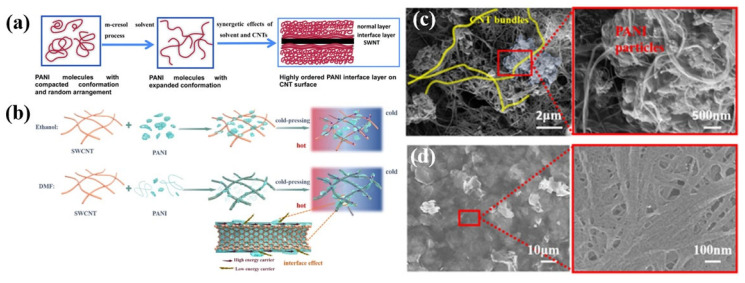
(**a**) Schematic representations of PANI layer on the surface of CNTs [47]; (**b**) Schematic of the microstructure of solvent-treated PANI/SWCNTs films: ethanol and DMF solvent; SEM images of PANI/0.9CNTs composite films with (**c**) ethanol and (**d**) DMF solvent treatment [66].

To date, there are generally two methods for preparing PANI/CNTs composites: physical blending [58,63] and in-situ chemical synthesis [64]. Physical blending refers to mixing polymers, fillers, and dopants to prepare composites. Wang et al. [65] reported the simultaneous improvement of *σ* and *S* in CNT-filled PANI composites. They showed that the surface of PANI-CSA with S/DWCNTs was smoother compared to that of PANIeb with S/DWCNTs, probably ascribing to the CSA doping effect which enhanced adhesion between the CNTs and PANI. PANI-CSA acts as a conductive bridge at the junctions between CNTs, promoting charge transfer and thus enhancing TE performance. With 66.7 wt% of S/DWCNTs, the PANI-CSA/S/DWCNTs composites exhibited higher PF of 34 μW m^−1^K^−2^. To further improve the power factor, more conductive DWCNTs were incorporated instead of S/DWCNTs. With the addition of highly conductive DWCNTs, the carrier mobility was increased significantly accompanied by decreased carrier concentration, resulting in a simultaneous increase in *σ* and *S*. When 30 wt% DWCNT was added, the *σ* of the PANI/CNT composite reached 610 S cm^−1^, *S* was 61 µV K^−1^ and maximum PF of 220 µW m^−1^K^−2^ was achieved at room temperature. Yao et al. [47] prepared PANI/SWCNT films by simply mixing CSA-doped PANI and SWCNTs in the m-cresol solution. The strong interaction between the m-cresol solvent and PANI can overcome the van der Waals interactions between PANI molecules, allowing PANI to shift from a compact to an expanded conformation. Moreover, SWCNT induced an ordered arrangement of PANI chains with expanded conformation on the SWCNT surface, forming a more ordered PANI interfacial layer and thus higher TE performance (Figure 3a). Ultimately, the maximum values of *σ* and *S* of the hybrid films reached 769 S cm^−1^ and 65 µV K^−1^, respectively. The maximum PF was 176 µW m^−1^K^−2^ and the ZT value was 0.12 at room temperature. 

Feng et al. [66] fabricated PANI/SWCNTs composite films through a vacuum filtration method by mixing SWCNTs dispersion with PANI in different solvents, in which the interfacial morphology can be controlled. With ethanol as the solvent, many isolated clusters of PANI appeared on the SWCNTs surfaces due to the poor solubility of PANI in ethanol (Figure 3b–d). When the ethanol solvent was replaced by DMF, PANI was uniformly coated on the surfaces of SWCNTs bundles, forming a large number of interfaces (CNTs-CNTs, PANI (film)-CNTs, PANI (particle)-CNTs), as shown in Figure 3b. This morphology contributes to the electron transfer in the conductive network and enhances the interfacial effect to improve the *σ* and *S* of the composite films. A further cold-press treatment made the PANI particles and SWCNTs composite denser, and the compact network structure contributed to high carrier concentration and high mobility. The combination of solvent and cold-pressure treatment promoted enhanced interfacial adhesion and strength, leading to prominent TE performance. Further temperature-dependence changes in the TE properties were conducted, which exhibited decreased *σ* and enhanced *S* with the increase of temperature within 300−420 K. Consequently, the PANI/0.9CNT composite film prepared by DMF solvent reached a maximum PF of 84.1 and 402 µW m^−1^K^−2^ when the temperature was 303 and 407 K, respectively. 

Wu et al. [67] investigated the influence of different polymerization times of PANI on the film formation and TE properties of PANI/SWCNTs composites which were obtained by mixing PANI and SWCNTs. With increasing the polymerization time, the film formation and flexibility of PANI were improved. The highest conductivity of 360 S cm^−1^ was obtained for PANI films with a polymerization time of 12 h. It’s interesting that although the molecular weight, structural morphology, and TE performance of pure PANI depend on the polymerization time of PANI, it had no significant effect on the TE performance of the PANI/SWCNTs composites with fixed SWCNTs content of 60 wt%. The moderate addition of SWCNTs enables the PANI and SWCNTs to form a network with the interlayer regions connected, allowing the off-domain carriers to spread throughout the network and the electrons to propagate rapidly along the π-π conjugated ordered regions. Consequently, with proper modulation of the mass fraction of SWCNTs and performed temperature, the maximum PF reached 100 µW m^−1^K^−2^ for PANI5h/0.6CNT at 410 K, and the composite film exhibited good environmental and structural stability. 

**Figure 4:**
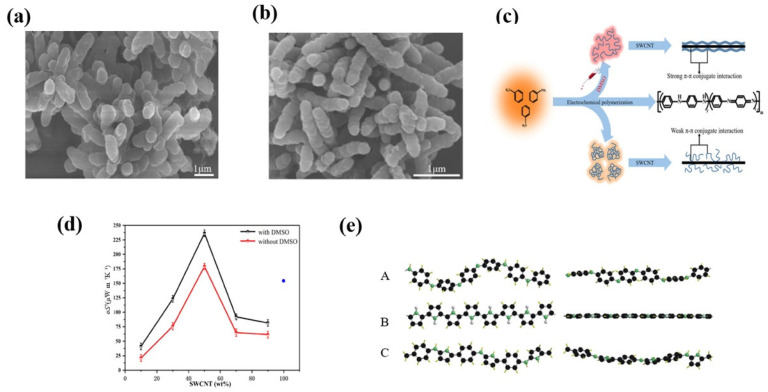
SEM images of (**a**) PANI and (**b**) PANI-DMSO [70]; (**c**) Schematic representation of the interface effects between PANI and SWCNT [70]; (**d**) Effect of the SWCNT content on the PF of PANI/SWCNT and PANI-DMSO/SWCNT composite films [70]; (**e**) Top view (left) and side view (right) of the partial chain segment conformation of PANi (A), PPy (B), and PANiPy (C) under the lowest energy state [72].

Furthermore, the three-electrode electrochemical polymerization method was also employed to synthesize high-performance PANI which was then physically mixed with CNTs [68,69]. Yin et al. [70] found that the diameter of PANI nanorods was much smaller with the addition of DMSO, as shown in Figure 4a,b. The addition of DMSO promoted PANI chains to change from coil-like to expand the structure, which improved the orderliness of PANI molecules [31,71] and enhanced the π-π interactions between PANI and SWCNTs. Therefore, a good conductive network was generated by the interfacial interactions and the ordered arrangement of PANI molecules, which facilitated carrier migration and thus improved the conductivity of the material (Figure 4c). The maximum PF of PANI-DMSO/SWCNTs reached 236.4 ± 5.9 µW m^−1^K^−2^ at room temperature when the concentration of SWCNTs was 50 wt% as shown in Figure 4d.

In addition, the alignment of the PANI chains can also be promoted by the incorporation of pyrrole units, in which a strong interaction was formed between the pyrrole and the aniline units (Figure 4e). Wang et al. [72] synthesized poly(aniline-co-pyrrole) random copolymer (PANiPy) with nanostructured morphology, which can be uniformly dispersed in the SWCNTs bundles within PANiPy/SWCNTs composites. Consequently, a maximum TE power factor of 98.5 μW m^−1^K^−2^ was achieved for 50 wt% PANiPy/SWCNTs composites at 430 K, higher than that of PANi/SWCNT, PPy/SWCNT, and PANi/PPy/SWCNT composites.

### 3.2. In-Situ Polymerization

#### 3.2.1. As-Prepared Composites

In-situ polymerization which enables PANI coatings to grow along CNTs at a molecular level has become one of the best methods for uniformly dispersing CNTs into a PANI matrix and enhancing interactions [73,74]. After polymerization, some PANI/CNT composites were employed as-prepared, and PANI components were mainly doped with hydrochloric acid (HCl) [64,75,76,77]. Meng et al. [78] reported PANI/MWCNTs composites via in-situ polymerization of PANI on highly conductive MWCNTs webs. First, a separate MWCNTs web consisting of entangled individual CNTs and CNTs bundles was fabricated as the matrix. After that, aniline was in-situ polymerized on the surface of MWCNTs to produce PANI/MWCNTs composites using HCl as the dopant. The *S* and PF of the composite were significantly improved with PANI coating, and the maximum PF value reached 5.0 µW m^−1^K^−2^ at 300 K. At the same time, the composite also possessed a lower thermal conductivity of about 0.45 W m^−1^K^−1^. Furthermore, Yao et al. [79] prepared PANI/SWCNTs composite films by in situ polymerization using HCl as the dopant. PANI grew along the SWCNT surface to form an ordered PANI molecular chain structure, promoting the increase of carrier mobility. With optimizing SWCNTs content, the maximum PF of the composite (41.4 wt% SWNT/PANI) reached 20 µW m^−1^K^−2^ with a *σ* of 125 S cm^−1^, and the corresponding ZT value was 0.004 at room temperature.

Apart from chemical oxidative polymerization, electrical polymerization is another commonly adopted technique to synthesize PANI and PANI/CNTs composites. The electric polymerization process can avoid the adsorption of impurities such as oxygen, into the interstices between the composite particles and the matrix material, resulting in superior microstructure and properties of the composites. Liu et al. [80] investigated the effect of the pulsed polymerization parameters on the film morphology and TE properties of PANI/MWCNTs composites. When relaxation time (t_2_) < 3 s, the PANI chains were thick, short, and had lateral growth. When t_2_ > 3 s, the aspect ratio of the PANI chains increased due to the oriented growth. The orientation arrangement of the long PANI chains facilitates the diffusion of MWCNTs, which can be replenished by the consumed MWCNTs and embedded in the PANI film in time during the next pulse cycle. Therefore, with the extension of t_2_, the time for MWCNTs to diffuse becomes longer. The overall trend of decreased electrical resistance means that the fibers grow along the chain direction, which is a result of the increased *σ* of the film. It had been shown that appropriate pulse parameters (pulse height (ϕ_1_) = 0.75 V, t_2_ = 3 s) could improve the *σ* and PF of the composite at room temperature.

Additionally, other PANI/CNTs composites with protonation of PANI by boric acid [81], 5-sulfosalicylic acid (SSA) [82,83], and CH_3_SO_3_H [84], have also been developed to modulate the microstructure and TE performance, promoting the improvement of PANI/CNTs composites for the TE applications.

#### 3.2.2. Secondary Doping Modulation

Currently, the secondary doping of camphor sulphonic acid (CSA)/m-cresol solutions is considered to be one of the most effective ways to improve the TE properties of PANI-based composites. Due to the strong Van der Waals gravitational forces between the functional groups in the polymer chain, when the CSA-doped PANI is dissolved in m-cresol, the carbonyl group in the CSA forms a hydrogen bond with the hydroxyl group of the m-cresol. The formation of a hydrogen bond between CSA and m-cresol may lead to a further separation of the positive ion (imine nitrogen in PANI chains) from the negative ion (CSA^−^), which increases the electrostatic repulsion between the positive imine nitrogen ions within the PANI chains. This internal force could conquer the Van der Waals gravitational force in the polymer chains and pull the conformation of the PANI chains from a “compaction coil” to an “expanded coil” [37,85,86,87,88,89]. With increasing m-cresol content to an extent, the surface of PANI film became homogeneous and smooth. Meanwhile, the ordered regions of the PANI molecular structure were enhanced, contributing to lower hopping barriers and increased carrier mobility. Finally, *σ* was increased significantly, and *S* remained constant or increased slightly [89].

Furthermore, doping level plays a vital role in improving the TE properties of PANI/CNTs composites. Generally, the *σ* of PANI is improved with increasing doping level to a certain extent, while *S* deteriorates owing to increased carrier concentration. Thereby, the maximum PF of PANI was achieved with a doping level of around 50%, which is commonly adopted to fabricate PANI-based composites [41]. Wang et al. prepared PANI/SWCNTs composite films using solvent treatment in combination with in-situ polymerization [90]. They demonstrated that m-cresol secondary doping and π-π interactions between PANI and SWCNTs can facilitate the formation of ordered PANI regions and promote carrier transport.

**Figure 5:**
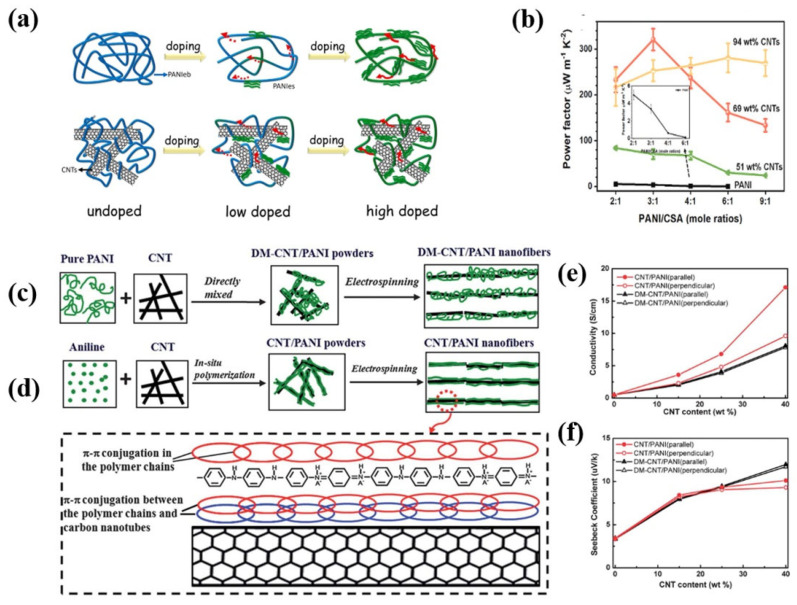
(**a**) Schematic diagram of pure PANI and PANI/CNTs composites with varying doping levels; (**b**) PF of PANI/CNTs with varied mole ratios of PANI: CSA, inset is enlarged PF of pure PANI [91]; Schematic representations of DM-CNT/PANI (**c**) and CNT/PANI (**d**) nanofibers, the enlarged part shows the highly ordered PANI chains on the surface of the CNTs; The electrical conductivity (**e**) and Seebeck coefficient of (**f**) DM-CNT/PANI and CNT/PANI nanofiber pellets as a function of CNT contents in parallel and perpendicular to the fiber axis [92].

Li et al. [91] investigated the influence of doping level on the microstructure and TE properties of PANI/SWCNTs films by adjusting the molar ratio of PANI/CSA. As the doping level decreased, the PANI chains were shifted from quinone to benzene units and electron transport deteriorated, resulting in decreased *σ*. However, due to the strong π-π interactions between PANI and SWCNTs, the addition of SWCNTs promoted the ordered orientation of PANI to facilitate carrier transport (Figure 5a). In contrast to pure PANI, the PF value was increased to 321 ± 21 µW m^−1^K^−2^ at a PANI/CSA molar ratio of 3:1 with SWCNTs loading of 69 wt%, higher than 234 µW m^−1^K^−2^ with PANI/CSA molar ratio of 2:1 as shown in Figure 5b. This could be due to the conductive network of SWCNTs providing additional pathways for carrier transport, as well as strong π-π interactions and the doping effect of SWCNTs, which promoted the ordering of PANI chains.

Wang et al. [92] prepared PANI/MWCNT composites by a combination of in-situ polymerization and electrostatic spinning, which successfully achieved highly oriented and ordered arranged PANI chains at the molecular level (Figure 5c,d). Due to the π-π interactions between PANI and CNTs, the orderliness of the PANI chains was initially improved during in-situ polymerization. Then it was further improved by electrostatic spinning treatment, where the electric field effect caused the PANI/CNT hybrid structure to align in parallel along fibers. The ordered molecular arrangement not only reduces the π-π conjugation defects in the polymer backbones caused by ring distortion, but also increases the effective degree of carrier delocalization, which improves the carrier mobility and enables the anisotropic TE properties. With the CNT content of 40 wt%, maximum TE performance was achieved with *σ* and *S* in the parallel direction of 17.1 S cm^−1^ and 10 µV K^−1^ at room temperature, respectively (Figure 5e,f).

#### 3.2.3. Modulation of the Polymerization Process

Carrier mobility is greatly influenced by the conformation of the polymer. PANI chains with extended coils of benzene conformation are more favorable for carrier transport [93,94,95], resulting in a highly ordered arrangement of PANI chains and thus highly conductive PANI/CNTs. Sobha et al. [96] prepared PANI/functionalized multi-walled carbon nanotubes (FMWCNTs) composites using conventional in situ polymerization and dynamic interfacial polymerization methods. The composites synthesized by in situ polymerization exhibited a thick core-shell structure with many protrusions. In contrast, the interfacial polymerization could avoid secondary growth, inducing a very homogeneous PANI coating with a thinner core-shell structure. The polymer chains formed an ordered molecular structure on the surfaces of the FMWCNTs, which increased carrier mobility. Consequently, *σ* of 960 S m^−1^ and *S* of 27.9 µV K^−1^ were obtained at room temperature.

In addition, Wang et al. [97] investigated the effect of polymerization temperature on TE properties of PANI/MWCNT composites by in-situ polymerization at room temperature and ice bath temperature. The ice-PANI (i-PANI)/MWCNTs were more compact than the room-PANI (r-PANI)/MWCNTs, and the CNTs were better wrapped by the PANI and well dispersed in the PANI. When the polymerization temperature was lowered to 0 °C, PANI was transformed from quinone to benzene rings, and the conformation was further expanded. i-PANI/MWCNTs composites exhibited a more ordered molecular arrangement and higher carrier mobility than r-PANI/MWCNTs, resulting in higher *σ*. Therefore, different polymerization processes have a great influence on the microstructure of PANI and electrical conductivities, the optimization of the polymerization process is crucial to modulate the TE properties of PANI/CNTs composites [98,99,100].

## 4. Post-Treatments

**Figure 6:**
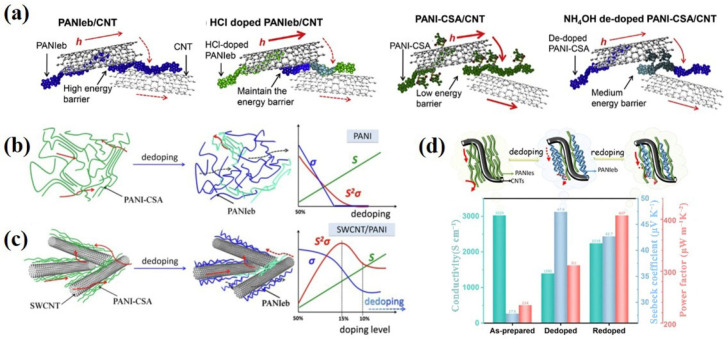
(**a**) Illustration of proposed carrier transport at CNTs-PANI-CNTs junctions for four composite films [101]. Scheme of carrier transport and TE properties for PANI-CSA films (**b**) and SWCNTs/PANI composite films (**c**) with the dedoping process [102]; (**d**) Schematic and TE performance diagram of a sequential dedoping-redoping strategy to modulate PANI/CNTs composite films [103].

Apart from the modulation strategies during composites preparation, the microstructure and carrier transport of PANI/CNTs composites can also be appropriately adjusted by post-treatments, such as the dedoping process, redoping approach, and so on. Wang et al. [101] proposed a facile gas treatment to regulate the interface of conjugated carbon structures and TE performance of PANI/(S/DW)CNTs composite films (Figure 6a). With HCl gas doping, the undoped polyaniline (PANIeb)/CNTs composite film exhibited a significant improvement in *σ* (700% increase) and a much high *S* (~90% value prior to the gas doping). This is mainly due to the existence of less affected PANIeb which is sandwiched between CNTs during HCl doping. A high energy barrier between PANI and CNTs could be maintained, contributing to relatively high *S*. Theoretical studies demonstrated that the PF value of PANIeb/CNTs composite films reached an optimum value when the energy barrier was 0.16 eV. This result indicated that the energy filtering approach induced by designing the energy barrier between PANI and CNTs could be an effective way to enhance the TE performance.

Li et al. [102,103,104] reported solvent post-treatment such as ethanol and ammonium hydroxide treatment, to adjust the doping level of PANI/SWCNTs composite films. With the partial removal of CSA content via solvent soaking treatment, the structure of PANI chains was changed from benzene to quinone, accompanied by deteriorated carrier transport along PANI chains (Figure 6b,c). However, with the addition of SWCNTs, the existence of a three-dimensional conductive CNTs network provided extra pathways to promote effective charge transport within the composite. This resulted in relatively high conductivity. In addition, the energy barrier between PANI and SWCNTs was enhanced after partial dedoping, which filtered out low-energy carriers. This induced a decreased carrier concentration, and contributed to greatly increased *S*. Ultimately, PF of the PANI/SWCNTs composite was significantly improved via proper modulation of the dedoping process. Overall, the final PF values of the ethanol and ammonium hydroxide-treated PANI/SWCNT composites were improved from 234 µW m^−1^K^−2^ to 362 µW m^−1^K^−2^ and 345 µW m^−1^K^−2^ at room temperature, respectively.

Apart from that, they have also proposed a sequential dedoping-redoping strategy to modulate the interfacial microstructure of PANI/SWCNTs composite films (Figure 6d). Firstly, the PANI/SWCNTs films were dedoped by immersing in ammonium hydroxide solution, which resulted in significantly decreased *σ* accompanied by increased *S*. The subsequent redoping process with HCl enhanced the doping level of PANI, with the expanded conformation of PANI chains, contributing to greatly improved *σ.* While *S* maintained relatively high, ascribing to the multiple interface structure in the PANI composite. This was probably generated from the incomplete dedoping and redoping process. Ultimately, a high PF of 407 µW m^−1^K^−2^ was obtained, which is one of the highest reported PF for PANI/SWCNTs composites at room temperature.

## 5. Multi-Components Hybrid Composites

**Figure 7:**
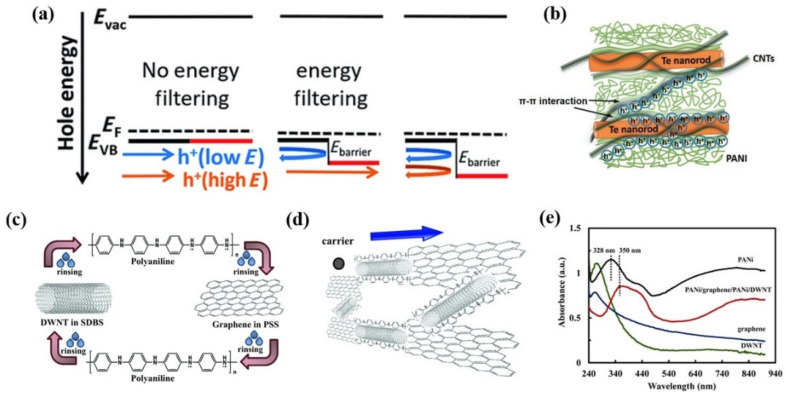
(**a**) Scheme of carrier energy filtering [105]; (**b**) Schematic illustration of interfacial interactions [23]; Schematic representation of the layer-by-layer (LbL) self-assembly procedure (**c**) and carrier transport in the PANI/graphene/PANI/DWNT nanocomposite (**d**) [109]; (**e**) UV–vis spectra of aqueous DWNT, graphene, PANI solutions, and PANI/graphene/PANI/DWNT film [109].

Due to the different energy levels, an energy barrier (E_barrier_) would be generated at the interfaces between polymer and filler, and the higher-energy carriers can be preferentially passed through, while the lower-energy carriers would be filtered out (Figure 7a) [105,106]. As low-energy carriers are detrimental to the *S*, the remaining high-energy carriers can enhance *S*, but the carrier concentration may be reduced when low-energy carriers are blocked [107]. According to the equation: PF *= S^2^σ*, the *S* has a greater effect on the PF than *σ*. Therefore, it is possible to enhance the energy filtering effect at the polymer/filler interface by creating composites with multiple interfaces [108], thus enhancing the *S* and PF of the composite. Currently, other components have been incorporated into the PANI/CNTs composites to enhance the energy filtering effect, developing high-performance multi-components TE composites.

Based on the study of Te/PANI hybrid films, Wang et al. [23] adopted MWCNTs to partially replace the Te nanorods to enhance their TE performance by properly tuning the interface of the composite (Figure 7b). In this ternary hybrid nanomaterial system, energy barriers at the Te/PANI and PANI/MWCNTs interfaces were 0.22 eV and 0.05 eV, respectively, which filtered out lower-energy carriers. The multiple interfaces within the constituents enhanced the energy filtering effect, and thus contributed to the increase of *S*. Moreover, the Te nanorods and MWCNTs can be served as a template to facilitate carrier transport. With optimal composition, PF of 54.4 µW m^−1^K^−2^ was obtained for 52 wt%Te-16 wt% MWCNTs/PANI film. Moreover, the incorporation of titanium oxide (TiO_2_) inorganic nanoparticles into A-CNT/PANI composites was reported by Erden et al. [110]. The TiO_2_/A-CNT/PANI ternary system showed higher *S* and higher PF compared with A-CNT/PANI composites. This result was attributed to the multiple energy barriers at multiple interfaces constructed by A-CNT, PANI, and TiO_2_, which induced an enhanced energy filtering effect to filter out low energy carriers and thereby the enhancement of *S*. The maximum PF reached 114.5 µW m^−1^K^−2^ for 30 wt% TiO_2_-70 wt% (A-CNT(70 wt%)/PANI(30 wt%)) ternary composites by subsequent water treatment at 40 °C to remove excess dopant (CSA).

An et al. [111] prepared a high-performance Au-doped PANI/CNTs network by embedding PANI into Au-doped CNT webs. The *S* and *σ* of the porous CNT webs increased after Au modification. While after PANI was embedded in the Au-doped CNT webs, the *π-π* conjugation interactions caused the PANI backbones to be orderly arranged, and the interfacial energy filtering effect led to an increase in the *S* of the composite system. Moreover, the thermal conductivity of the composites was greatly decreased, resulting in a *ZT* of 0.203 for Au-doped PANI/CNTs webs at room temperature.

In addition, the PANI/graphene/PANI/DWNT polyelectrolyte carbon nanocomposites (PCNs) were prepared by Cho et al. [109] using the layer-by-layer assembly method, as shown in Figure 7c. The PCNs with 40 layers of PANI/graphene/PANI/DWNT showed *σ* of 1080 S cm^−1^ and *S* of 130 µV K^−1^, with a corresponding maximum PF of 1825 µW m^−1^K^−2^. Their excellent TE performance was attributed to the 3D conjugated network structure formed by the three components. The PANI-coated DWNT served as a bridge to connect adjacent graphene sheets, as shown in Figure 7d. As more layers were deposited, the *σ* of the nanocomposite films was enhanced because of the increased density of connections between the polymer and carbon nanoparticles in the layers. UV-vis spectrum showed that the benzene peak at 328 nm was red-shifted for PANI deposited on DWNT (Figure 7e), indicating a conformation change of PANI from a random (ring twist) to an expanded chain with an increased degree of electron delocalization. This extended chain conformation facilitated better carrier transport and thereby enhanced *σ*.

Consequently, to pursue the high TE performance of organic composites, an energy filtering effect that filters low-energy carriers would be an efficient approach to enhancing *S* through the rational design of interfaces and energy barriers. On this basis, with appropriately adjusting carrier mobility and carrier concentration, *σ* can be maintained at a relatively high level accompanied by enhanced *S*, leading to high TE PF ultimately.

## 6. Electronic Type and Anisotropy of CNTs

**Figure 8:**
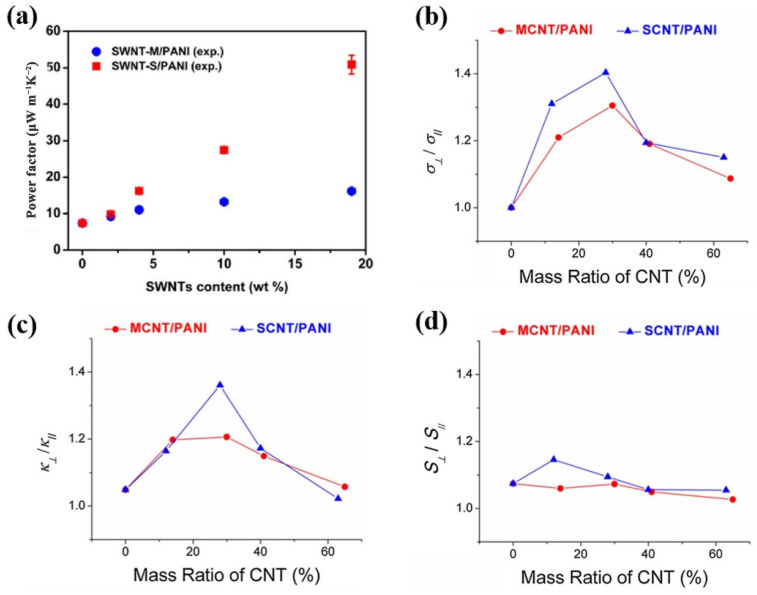
(**a**) Power factor of SWNT-M/PANI and SWNT-S/PANI composite films with varied SWNTs content [63]; Anisotropy properties of MCNT/PANI and SCNT/PANI composites measured perpendicular and parallel to the cold pressing pressure axis for (**b**) *σ*, (**c**) *k,* and (**d**) *S* [112].

In addition to the above-mentioned reports, this section discussed the impact of other factors on TE performance. Wang et al. [63] investigated the effect of the electronic type of SWCNT (metallic single-walled carbon nanotubes (SWNT-M) and semiconducting single-walled carbon nanotubes (SWNT-S)) on the TE properties of PANI/SWCNT composite films. They found that SWNT-S exhibited a higher *S* than SWNT-M, while the *σ* difference was negligible. This could be due to the strong energy filtering effect at the interface between SWNT-S and PANI, which could effectively filter low-energy carriers while allowing high-energy carriers to pass through. The resultant PF of SWNT-S/PANI was 3 times higher than that of SWNT-M/PANI composite film with 19 wt% SWNT at room temperature, as shown in Figure 8a.

Furthermore, due to the anisotropy of the CNTs arrangement, PANI/CNTs composites exhibit anisotropy in their TE properties [113,114]. Chen et al. [112] showed that CNTs content had a large effect on the anisotropy of the TE properties, as shown in Figure 8b–d. At 400 K, the anisotropy in the *k* and *σ* was greatest when the CNTs content is 30 wt%. The *k* perpendicular to the cold pressing direction (*κ_⊥_*) was 30–40% higher than that parallel to the cold pressing direction (*κ_//_*), while the anisotropy in the *σ* was about 40–50% higher. When further increasing the content of CNTs, the anisotropy of PF and *ZT* values of the PANI/CNTs composite became inconspicuous. This is due to the presence of numerous agglomerated carbon nanotubes, which were not effectively coated by PANI. It would reduce the orientation of CNTs in the direction perpendicular to the pressure, and the ordered alignment of PANI on the CNTs surface was also reduced, resulting in reduced anisotropy in TE properties of PANI/CNTs composites.

## 7. Summary and Outlook

In summary, different methods such as the addition of surfactants, mechanical stirring and grinding, and CNTs functional group modification were discussed to reduce the agglomeration of CNTs and better disperse the CNTs. The homogeneous CNTs dispersion promotes the formation of conductive CNTs networks and facilitates carrier transport, contributing to the great enhancement of the TE performance. Subsequently, versatile PANI/CNT composites have been prepared mainly by physical blending and in-situ polymerization, and the composites can be used directly or after secondary doping. The microstructure of the composites can also be regulated by adjusting the polymerization parameters of PANI. Moreover, post-treatment methods such as solvent treatment and adjustment of doping levels were also discussed as effective strategies to optimize carrier transport. In addition, the addition of other components to the composites can enhance the energy-filtering effect, further improving the TE properties. 

This paper summarized the recent development of versatile PANI/CNTs composites, which have attracted tremendous interest for TE applications (Table 1). Various approaches to improve the TE performance of PANI/CNTs composites in recent years were comprehensively discussed. Generally, higher doping levels lead to higher *σ* but may be accompanied by a decrease in *S*. At the microscopic level, the ordering of molecules within the composites and interfacial effects are important factors that affect carrier conduction. To further improve the TE properties of PANI/CNTs composites, several pending problems need to be solved:

(1) There is still a large gap in preparing high-performance PANI/CNTs composites with low CNTs content, due to the high cost of CNTs and easy agglomeration with high CNTs content;
(2) How to promote the extended conformation of PANI molecules and induce their alignment neatly on the surface of CNTs to form a more ordered PANI interfacial layer, thus increasing the carrier mobility and TE performance of PANI/CNTs composites;
(3) Novel approaches to achieving high *σ* and *S* synergistically, promoting TE efficiency of PANI/CNTs composites.

## Figures and Tables

**Table 1:** A summary of the TE properties of some typical PANI/CNTs composites.

Strategies			*σ* (S cm^−1^)	*S* (μV K^−1^)	*PF* (μW m^−1^K^−2^)	Ref.
Dispersion strategies of CNTs		PANI/MWNT (ρ_SDS_ = 0.182M)	14.1	79.8	8.98	[57]
		PANI/MWNTs	1.59	~26	~0.107	[58]
		PANI/PPy-MWNT	30.34	31.2	3.1	[62]
		PANI/A-CNT	1871	46.5	401	[59]
Preparation engineering	Physical blend	PANI/DWCNTs	610	61	220	[65]
		PANI/SWCNT	769	65	176	[47]
		PANI/SWCNTs	1965	21.21	114.4	[66]
		PANI/SWCNTs	~4000	~19	100	[67]
		PANI/DMSO/SWCNT	842.5	53	236.4	[70]
		PANiPy/SWCNTs	423.2	51.5	98.5	[72]
	In-situ polymerization	PANI-HCl/MWCNTs	61.47	28.6	5.04	[78]
		PANI-H_2_SO_4_/MWCNTs	76.96	28.2	6.12	[80]
		PANI-HCl/SWCNTs	125	40	20	[79]
		PANI-CSA/MWNT	17.1	10	0.171	[92]
		PANI-CSA/SWCNTs	2898	33.3	321	[91]
		PANI-CSA/SWNTs	1440	38.9	217	[90]
		PANI/FMWCNTs	9.6	27.9	0.747	[96]
		i-PANI/MWCNT	21.6	12.8	0.355	[97]
Post-treatments		PANI/SWCNTs	~2356	39.2	362	[104]
		PANI/SWCNTs	2025	41.3	345	[102]
		PANI/SWCNTs	2238	42.7	407	[103]
Multi-components hybrid composites		PANI/Te-MWCNT	137	63	54.4	[23]
		PANI/graphene/PANI/DWNT	1080	130	1825	[109]
		PANI/a-CNT/TiO_2_	2183	22.9	114.5	[110]
		Au-PANI/CNT	1106	150.86	2517.1	[111]
Electronic type and anisotropy of CNTs		PANI/SWNT-S	~390	~36	51	[63]
		PANI/SWCNTs	~110	~42	~20	[112]

## Data Availability

Not applicable.
